# Joint clustering and prediction approach for travel time prediction

**DOI:** 10.1371/journal.pone.0275030

**Published:** 2022-09-23

**Authors:** Hima Elsa Shaji, Arun K. Tangirala, Lelitha Vanajakshi

**Affiliations:** 1 Department of Civil Engineering, Indian Institute of Technology Madras, Chennai, India; 2 Department of Chemical Engineering, Indian Institute of Technology Madras, Chennai, India; 3 Department of Civil Engineering / Robert Bosch Centre for Data Science and Artificial Intelligence Indian Institute of Technology Madras, Chennai, India; Chongqing Jiaotong University, CHINA

## Abstract

Modeling and prediction of traffic systems is a challenging task due to the complex interactions within the system. Identification of significant regressors and using them to improve travel time predictions is a concept of interest. In previous studies, such regressors were identified offline and were static in nature. In this study, an iterative joint clustering and prediction approach is proposed to accurately predict spatiotemporal patterns in travel time. The clustering module is tied to the prediction module, and a prediction model is trained on each cluster. The combined clustering and prediction are then iterated until a chosen metric is optimized. This orients clusters of data towards prediction while enabling model development on subsets of travel time data with similar prediction complexity. The clusters created using the joint clustering and prediction approach confirmed to the real-world traffic scenario, forming clusters of high travel time at busy intersections and bus stops across the study stretch and forming clusters of low travel time in the sub-urban areas of the city. Further, a comparison of the developed framework with base methods demonstrated a decrease in prediction errors by at least 22.83%. This indicates that creating clusters of data that are sensitive to the quality of predictions using the joint clustering and prediction framework improves the accuracy of travel time predictions. The study also proposes criteria for choosing the best predictions when cluster-based predictions are used.

## Introduction

Modeling of traffic systems is essential for the development of new road networks, analysis of existing ones, prediction of their future performance, and planning measures for improvements. The modeling of traffic systems is challenging due to their inherent complexities and associated uncertainties. Traffic systems are influenced by human, vehicle, road, and environmental characteristics and their interactions with each other. These characteristics make it difficult to identify suitable variables that can capture the temporal and spatial variations in the system.

Travel time is an important traffic variable that is widely used to characterize the traffic system. Travel time data exhibits both temporal and spatial variations; it may vary depending on the day, time, location, etc. The high variability in the system must be considered for accurate prediction of travel times. Earlier studies have predicted travel times by offline identification of significant regressors based on the assumption of fixed patterns [1]. However, these patterns may not be static and may depend on traffic fluctuations. Thus, there is a need to automatically identify the correct regressors by recognizing the natural groupings in the data for better modeling of the system.

While modeling complex systems, it is common to consider a set of local models and analyze the system in parts instead of using a global model. This technique is called multiple model learning [2]. Here, input partitions are estimated and models are built for each of the partitions. As the groups in the spatiotemporal data are unknown, supervised learning algorithms may not be an efficient tool to partition the data. Clustering-based partitioning can be used when there is no prior knowledge of the system and input space partitions are unknown [3]. Clusters can represent the system more accurately, and can thus improve prediction accuracy.

The choice of the prediction technique can be based either on process knowledge or the end-use. Historical averaging, linear regression, linear and non-linear time series models, and machine learning-based approaches such as Artificial Neural Networks (ANN), Support Vector Machines (SVM), and Deep Learning (DL) are commonly used for travel time predictions. The goal of the present study was not only accurate prediction of travel time, but also meaningful interpretation of data. Techniques such as historical averaging, linear regression, and time series modeling, although easier to interpret, could compromise the accuracy of predictions. On the other hand, with increase in accuracy, the model may lose transparency and interpretability of the results. For example, DL approaches may afford high accuracy at the expense of transparency.

Many studies have used clustering as a pre-tool for the prediction and estimation of traffic variables [4–7]. However, clustering and prediction modules are treated independently in these studies, and their interactions are not considered. In this study, the interactions between clustering and prediction were considered and utilized in improving the predictions. Prediction complexity was used as one of the metrics for clustering highly varying data and a prediction methodology that can use these data-driven groupings was developed. A joint clustering and prediction approach was proposed, in which, both clustering and prediction were tied together in an iterative procedure to improve the accuracy of prediction. The iterative method oriented data clusters towards prediction while enabling the development of models on clusters of data with similar prediction complexities.

## Literature review

The ability of ML approaches to solve complex and non-linear problems makes them a suitable prediction strategy for the prediction of traffic state variables. Artificial Neural Networks (ANN) is a popular ML technique used in predictions. [2, 8–16], have shown NN-based travel time predictions outperforming other predictions based on historical average, regression and Kalman filter.

Clustering has been used in the past as a pre-tool for the identification of significant regressors and the prediction of many traffic variables. Important clustering algorithms used for the prediction of traffic variables include the *k*-means clustering algorithm [17–24], hierarchical clustering algorithm [25–28], and Kohonen SOM [4, 29–31]. The earlier studies indicated that the performances of the models built on clustered data were better than traditional models. However, most of these studies were carried out under homogeneous traffic conditions. Few studies have used clustering to identify regressors in mixed traffic conditions due to the limited data availability. A few related studies on regressor identification under mixed traffic conditions by [1, 32] assumed fixed patterns to identify data patterns. The performances of *k*-means, hierarchical, and Kohonen SOM clustering algorithms in predicting bus travel times were compared by [33]. The *k*-means clustering was found to perform best.

The review of literature shows that clustering-based multiple model learning for data understanding and prediction have hitherto been explored largely for homogeneous traffic conditions. In addition, the clustering and prediction modules have been studied independently, without considering their effects on each other. The question arises whether clusters can be formed such that they improve the prediction accuracy. [34] used a prediction error-based clustering approach for data generated using multiple models and different sampling conditions. Prediction errors were iterated until the clustering procedure with modified model orders contained only significant variables. However, the interpretability of the clusters formed was not analyzed. It is not yet known if meaningful clusters that relate to real-world traffic conditions can be formed using better feature vectors and if such approaches would lead to better accuracy.

The study reported herein aimed to address these gaps in literature through the design and testing of a novel joint clustering and prediction framework. The reported approach created meaningful clusters of data that correspond to real-world traffic conditions and was sensitive to the quality of predictions. Another significant contribution of the study is that the proposed framework is agnostic to both the domain (prediction of travel times in this study) and the model used for prediction (ANN in this study). Thus, this umbrella framework can be applied to prediction exercises regardless of the domain and the prediction model used.

The developed joint clustering and prediction framework was applied to predict travel times on an urban arterial road. The study also systematically analysed the changes in the clusters across the iterative steps and the effect of feature vectors on prediction strategies. In addition, the predictions made by the framework were compared with those made using base data, data with fixed clusters and data-derived clusters to assess performance.

## Problem statement

A joint clustering and prediction approach was formulated, in which, clusters of data were identified, and accurate predictions of travel times were obtained using an iterative approach to minimize errors. Here, the input to the clustering algorithm was from the prediction module and vice versa. Let (*Y*_*t*,*s*_)_*k*_ denote the travel time of section *s* for trip *t* in the *k*^th^ cluster, where *t* = 1, 2, …, *T*; *s* = 1, 2, …, *S*, and *k* = 1, 2, …, *K* where *T*, *S*, and *K* are the total number of bus trips, the total number of sections across the study stretch and the optimum number of clusters, respectively. Given (*Y*_*t*,*s*_)_*k*_, the study aimed to predict the travel times of trips *t* = *T*+1, …, *T*+Δ*t*. Clusters were formed by partitioning the data such that *C*_1_ ∪ *C*_2_ ∪ … ∪ *C*_*K*_ = *Y*, the whole set of travel time measurements. The clustering algorithm aimed to find *K* clusters in the data such that the within-cluster sum of squares is minimized, as shown in Eq 1.

$$\begin{array}{c} \text{arg}\;\text{min}\sum_{k=1}^{K}\sum_{y_{i}\in C_{k}}\|y_{i}-\mu_{k}{\|}^{2} \end{array}$$ (1)

Here *y*_*i*_ is a point belonging to cluster *C*_*k*_ and *μ*_*k*_ is the mean of the points in cluster *C*_*k*_. The prediction algorithm, on the other hand, aimed to find the model parameters such that the prediction errors were minimized. Here, the objective function was to optimize the weights to minimize the loss function *L* for all the *n* data points, given by Eq 2.

$$\begin{array}{c} L=\frac{1}{n}\sum_{i=1}^{n}{\left(\hat{y}-y_{i}\right)}^{2} \end{array}$$ (2)

## Methodology

In this section, the three base methods, namely, predictions on base data, data with fixed clusters and data-derived clusters, and the proposed joint clustering and prediction framework are discussed.

### Base data

In this case, no groups or patterns were extracted from the data set. A single model was trained on the entire dataset and was used for prediction. The model was trained using the 500m section travel times of previous *n* trips that occurred before the trip under consideration. For this study, the value of *n* was chosen as ten, and predictions were made.

### Fixed clusters

In many earlier studies that reported prediction using pattern analysis, the data set was manually grouped using chronological factors for the same bus route [1, 32],. It was assumed that manual grouping based on fixed patterns could sufficiently separate the data set into distinct groups. To check the validity of this assumption, in this work, the data set was divided into fixed clusters manually. Here, three groups were considered—weekday peak, weekday off-peak, and weekend trips, assuming that these trip patterns were different from each other. Three separate models were then trained on these datasets.

### Data-derived clusters

In earlier studies that used clustering in prediction strategies, both clustering and prediction were kept as disjoint modules [2, 6]. In this work, the data set was first divided into an optimum number of clusters using a suitable clustering algorithm, and separate predictions were made on each of these clusters.

### Proposed joint clustering and prediction approach

**Fig 1:**
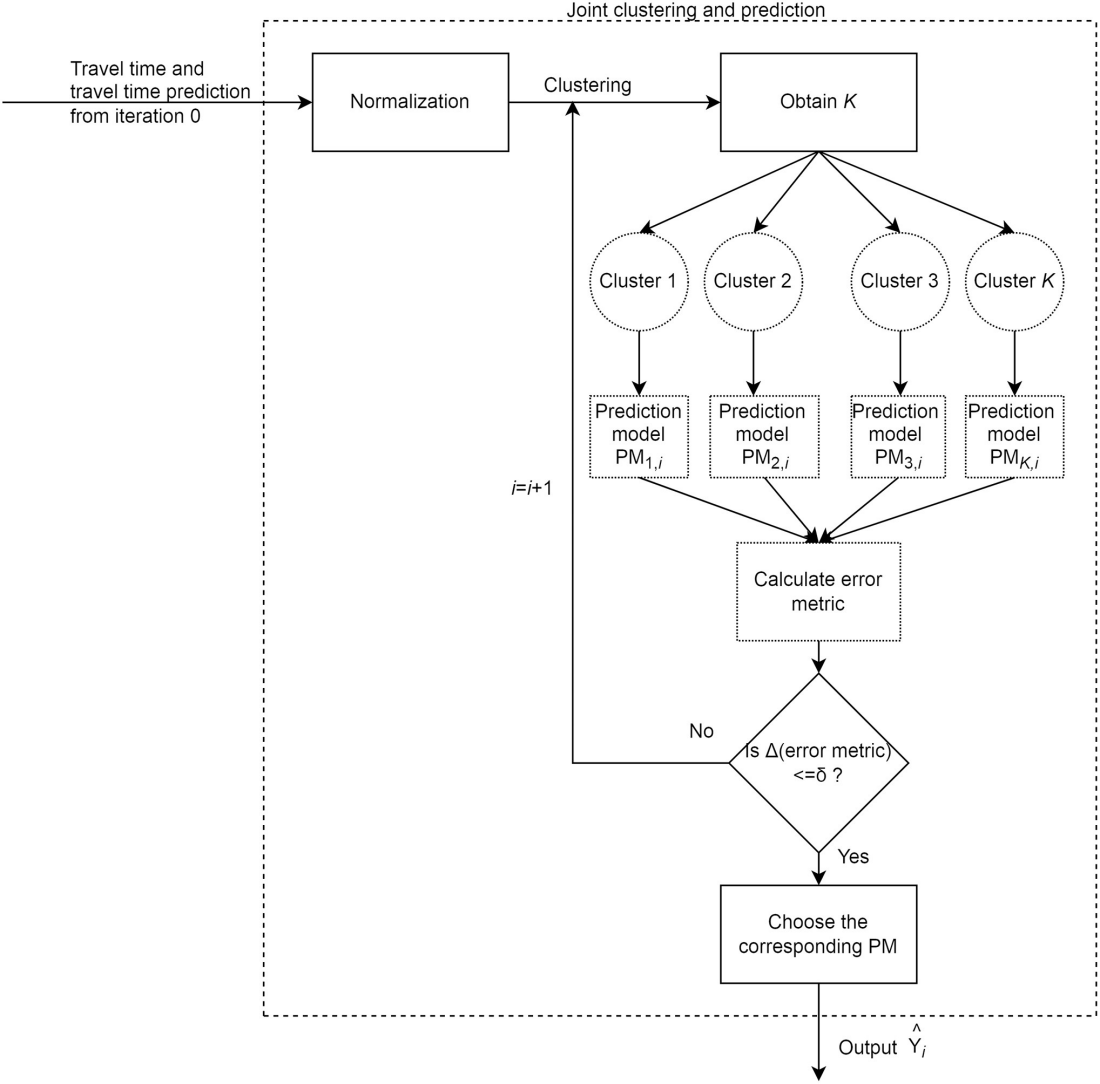
Joint clustering and prediction framework.

The proposed methodology used a novel joint clustering and prediction approach to obtain accurate predictions. Both clustering and prediction were combined in an iterative framework. The iterative procedure oriented clusters of data towards prediction while enabling the development of models on subsets of data with similar prediction complexity. Thus, clusters of data were oriented towards improving the prediction accuracy. Both travel time and travel time predictions were used as feature vectors for clustering to identify points that were similar in magnitude and predictability. Fig 1 shows the framework of the algorithm used in this study.

## Joint clustering and prediction

In this study, an iterative training procedure was chosen to train the predictive model, and the variation of cluster membership in each iteration was studied. The input space partitions were chosen such that a further increase in iterations did not reduce training prediction errors. The framework of the developed joint clustering and prediction approach is shown in Fig 1. The travel times and travel time predictions obtained in iteration 0 by training a single ANN on the entire data set were used as starting seeds. *k*-means clustering algorithm was used to group the feature vectors, and the optimum number of clusters was found using the Elbow method [35]. For every iteration *i*, the travel time values and travel time predictions from (*i*-1)^th^ iteration were used as feature vectors for clustering. The process was terminated once the error metric stabilized. This ANN was chosen as the best predictor and was used to predict the testing data set. The developed joint clustering and prediction approach is summarized in Table 1.

**Table 1:** Joint clustering and prediction.

**Input**: Set of travel times in training **Y**_(*t*×*S*)_, where *t*—number of trips in training, and *S*—number of sections across the study stretch. **Output**: *K* ANN models **Method**: 1. Intialize number of clusters, *k*_*in*_=1. 2. For every point in **Y**, find 3 neighboring points (*x*_1_, *x*_2_, *x*_3_) with similar day index *d*, 15-minute index *m*, section index *s*. 3. Train single ANN on entire **X** = [**x**_**1**_, **x**_**2**_, **x**_**3**_] where **x**_1_ = [*x*_11_, *x*_12_, …, *x*_1(*txS*)_]^T^, **x**_2_ = [*x*_21_, *x*_22_, …, *x*_2(*txS*)_]^T^, and **x**_3_ = [*x*_31_, *x*_32_, …, *x*_3(*txS*)_]^T^. 4. Using **X**, get travel time predictions $\hat{Y}$. **Repeat** 5. Use *Y* and $\hat{Y}$ as feature vectors for *k*-means clustering. 6. Find optimum number of clusters, *K* using the elbow method. 7. Train separate ANNs on each of the clusters. 8. Obtain updated travel time predictions $\hat{Y}$. 9. Calculate maximum MAPE of all trips. **Until**: Δ(MAPE) ≤ *δ*

On completion of training, the ANNs were used for testing. The challenge lay in associating a section travel time in the testing dataset to a specific cluster since the future was unknown. For example, for each test section travel time, *K* predictions could be obtained using each of the *K* ANNs. Choosing the appropriate prediction model from these *K* options was a challenging task. To address this, two approaches—a selection- based approach and a fusion-based approach were proposed.

The first approach, a selection-based criterion, involved the exploration of the features of the clusters, as shown in Table 2. This criterion was expected to work well for the low travel time clusters (say, cluster 1 and cluster 2 in our case) with higher memberships. For high travel time clusters (cluster 3 and cluster 4), this selection strategy may not work as they have lower membership. This resulted in the mapping of a high travel time section to a low travel time section. To overcome this, the predictions were refined by first labeling the section under consideration as high or low travel time section as shown in Table 3, and a revised selection criterion was proposed as shown in Table 4.

**Table 2:** Selection-based criterion.

**Input**: Set of *K* predictions $\hat{Y}=[{\hat{Y}}_{1},{\hat{Y}}_{2},\ldots ,{\hat{Y}}_{K}]$ for a test point from each of the *K* clusters. Section for which prediction is to be made (*s*_test_) Time at which prediction should be made (*m*_test_) **Output**: Best prediction for the test point, ${\hat{Y}}_{\text{best}}$ **Method**: 1. In each cluster *K*, find the number of matching points *N* : *s*_*i*_ = *s*_test_ and *m*_*i*_ = *m*_test_. 2. Find *c* = arg max(*N*) 3. Determine ${\hat{Y}}_{\text{best}}$=${\hat{Y}}_{c}$

**Table 3:** Classification into low and high travel time section.

**Input**: Sections along the study stretch (*s*_*i*_) **Output**: Classifying *s* into high (HT) or low travel time (LT) section **Method**: 1. For high travel time clusters, determine the proportion of members belonging to each section. 2. **IF** proportion(*s*_*i*_ ∈ high travel time cluster) < 0.2, **THEN** Assign *s*_label_=LT **ELSE** Assign *s*_label_=HT

**Table 4:** Revised selection-based criterion.

**Input**: Set of *K* predictions $\hat{Y}=[{\hat{Y}}_{1},{\hat{Y}}_{2},\ldots ,{\hat{Y}}_{K}]$ for a test point from each of the *K* clusters. Section for which prediction is to be made (*s*_test_) Time at which prediction should be made (*m*_test_) **Output**: Best prediction for the test point, ${\hat{Y}}_{\text{best}}$. **Method**: 1. In each cluster *K*, find the number of matching points *N* : *s*_*i*_ = *s*_test_ and *m*_*i*_ = *m*_test_. 2. **IF** (*s*_label_=LT) **THEN** Find *c* = argmax(*N*) Determine ${\hat{Y}}_{\text{best}}={\hat{Y}}_{c}$ **ELSE** Find weights *w*_*i*_ = [*N*_1_×*S*_1_, *N*_2_×*S*_2_, …, *N*_*k*_×*S*_*k*_], where *S*_*i*_ is the standard deviation of travel times in each of the *k* clusters. Find *c* = argmax(*w*) Determine ${\hat{Y}}_{\text{best}}={\hat{Y}}_{c}$

In the second approach, a fusion-based criterion was devised, in which, a weighted average of all the *K* predictions was used. The algorithm for the fusion-based strategy is provided in Table 5.

**Table 5:** Fusion-based criterion.

**Input**: Set of *K* predictions $\hat{Y}=[{\hat{Y}}_{1},{\hat{Y}}_{2},\ldots ,{\hat{Y}}_{K}]$ for a test point from each of the *K* clusters. Section for which prediction is to be made (*s*_test_) Time at which prediction should be made (*m*_test_) **Output**: Best prediction for the test point, ${\hat{Y}}_{\text{best}}$. **Method**: 1. In each cluster *K*, find the number of matching points *N* : *s*_*i*_ = *s*_test_ and *m*_*i*_ = *m*_test_. 2. Find weights *w*_*i*_ = [*N*_1_/*N*_1*T*_, *N*_2_/*N*_2*T*_, …, *N*_*K*_/*N*_*KT*_], where *N*_*iT*_ is the standard deviation of travel times in each cluster *K*. 3. Assign ${\hat{Y}}_{\text{best}}$ = $w_{1}\times {\hat{Y}}_{1}+w_{2}\times {\hat{Y}}_{2}+\ldots +w_{K}\times {\hat{Y}}_{K}$

## Data collection

The data used for the study were collected using the GPS units fixed on the Metropolitan Transport Corporation (MTC) buses in Chennai, the capital city of the state of Tamil Nadu, India. The 19B bus route was chosen as the study stretch. The route spans a length of 29.4 kms and connects Kelambakkam, a suburban area of the city, to Saidapet, a major commercial area of the city. The northbound 19B route was chosen for the analysis. The GPS data were collected every 5 seconds from the GPS units fitted in these buses. A total of 1,231 trips were collected for 45 days. The obtained GPS data included the date, timestamp, latitude, and longitude of the bus location. The distance between two consecutive GPS points was calculated using the Haversine formula [36]. For analysis, the route was divided into sub-sections, each of length 500 m, leading to 55 sections. The 500 m section travel times were calculated using interpolation.

## Implementation and results

The joint clustering and prediction approach was implemented, and travel times of 500 m sections were predicted using ANN. The prediction accuracy was quantified using Mean Absolute Percentage Error (MAPE), Mean Absolute Error (MAE), and Normalized Root Mean Square (NRMSE). 75% of the data points were used for training, and the remaining 25% were used for testing. The characteristics of the clusters formed, and the prediction performance were analyzed in detail and are presented in the following sections. The results from the joint clustering and prediction approach are then discussed, followed by a performance comparison with the base methodologies.

### Variation of MAPE and cluster memberships over iterations

**Fig 2:**
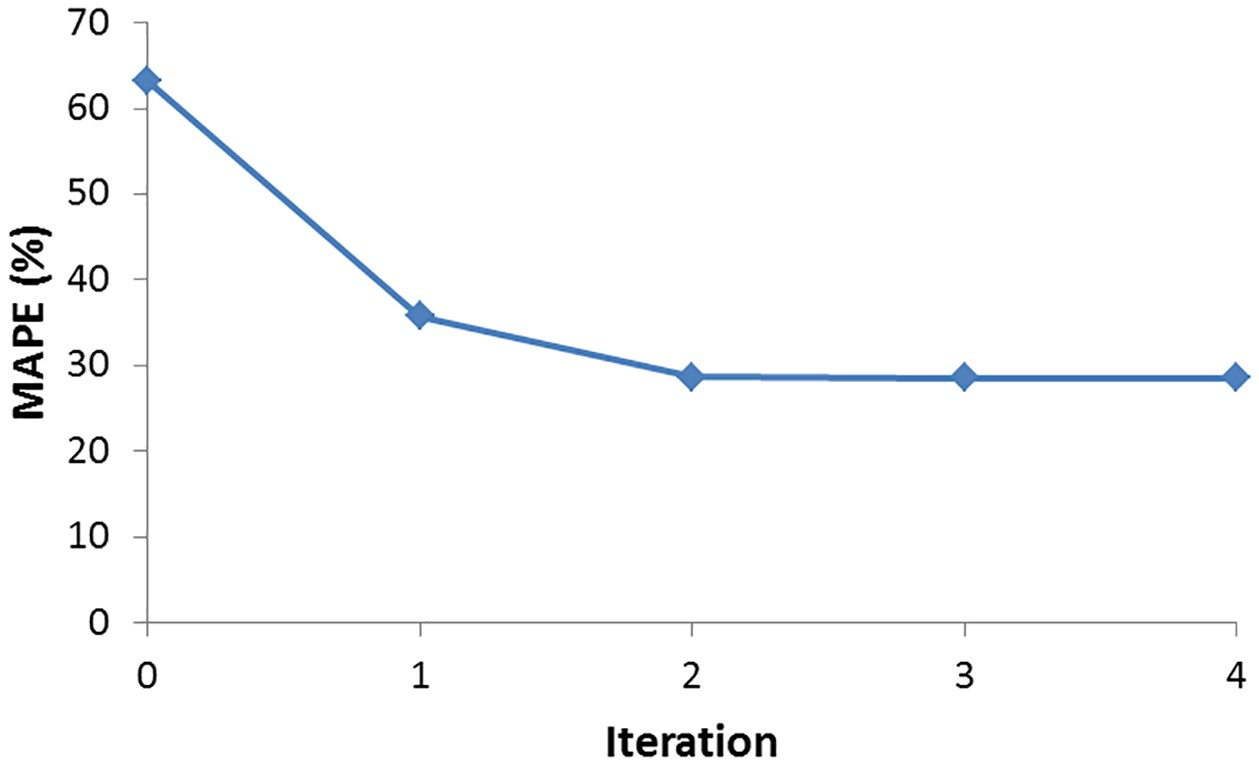
MAPE for maximum error trip over iterations.

In joint clustering and prediction, the variation of MAPE was used as the decision criterion to terminate the iterative training. The MAPE of the trip with the highest prediction error was plotted, and the iteration was terminated when the change in MAPE was negligible. Fig 2 shows the variation of MAPE over iterations. In the initial iteration 0, where a single model was trained on the entire dataset, the MAPE was high, and it decreased over iterations. This indicates the merit of training separate models on clusters than training a single model on the entire dataset. Furthermore, there was not much change in MAPE after the second iteration. To further analyze this phenomenon, the changes in cluster memberships over iterations were studied, and the membership details of each cluster formed are given in Table 6. The cluster memberships stabilized over iterations, and after the second iteration, there was not much change in the membership of the clusters. Let this iteration be denoted by *x*. Once the points similar in magnitude and predictability were grouped within a cluster, the change in MAPE and cluster memberships became minimal. The process was terminated after the *x*^th^ iteration. However, it must be noted that this case was only for this particular dataset. Any other dataset may require a different number of iterations to stabilize the MAPE and cluster memberships.

**Table 6:** Cluster memberships over iterations.

	Cluster 1	Cluster 2	Cluster 3	Cluster 4
Iteration 1	27069	19369	3614	713
Iteration 2	27638	18800	3614	713
Iteration 3	27643	18795	3614	713
Iteration 4	27644	18794	3614	713

### Variation of measured and predicted travel times over iterations

**Fig 3:**
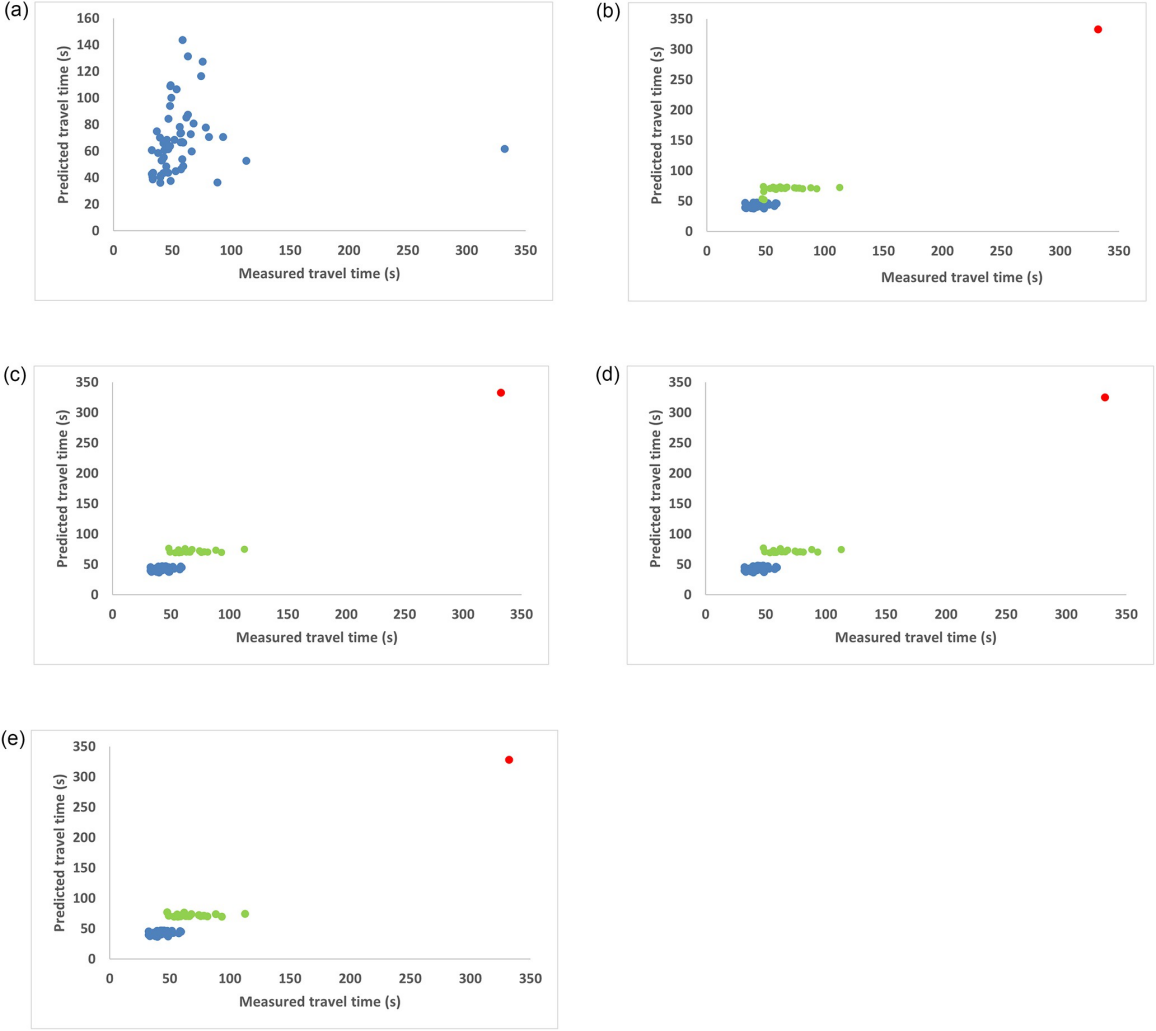
Measured vs predicted travel times over iterations. (a) Iteration 0, (b) Iteration 1, (c) Iteration 2, (d) Iteration 3, (e) Iteration 4.

Fig 3 shows the variation of measured and predicted travel times for a sample trip over iterations as the training progressed. Each color denotes the cluster to which the data point belongs, over iterations. In the initial iteration, iteration 0, a single model was trained on the entire dataset; that is, all the points were assumed to belong to a single cluster. From iteration 1, separate training models were trained on each of the clusters formed. The cluster memberships changed over iterations and stabilized around iteration 2, after which, there was no change in the cluster memberships. For the sample trip under consideration, the data points belonged to cluster 1, cluster 2, and cluster 4, and there was no data point in cluster 3.

**Fig 4:**
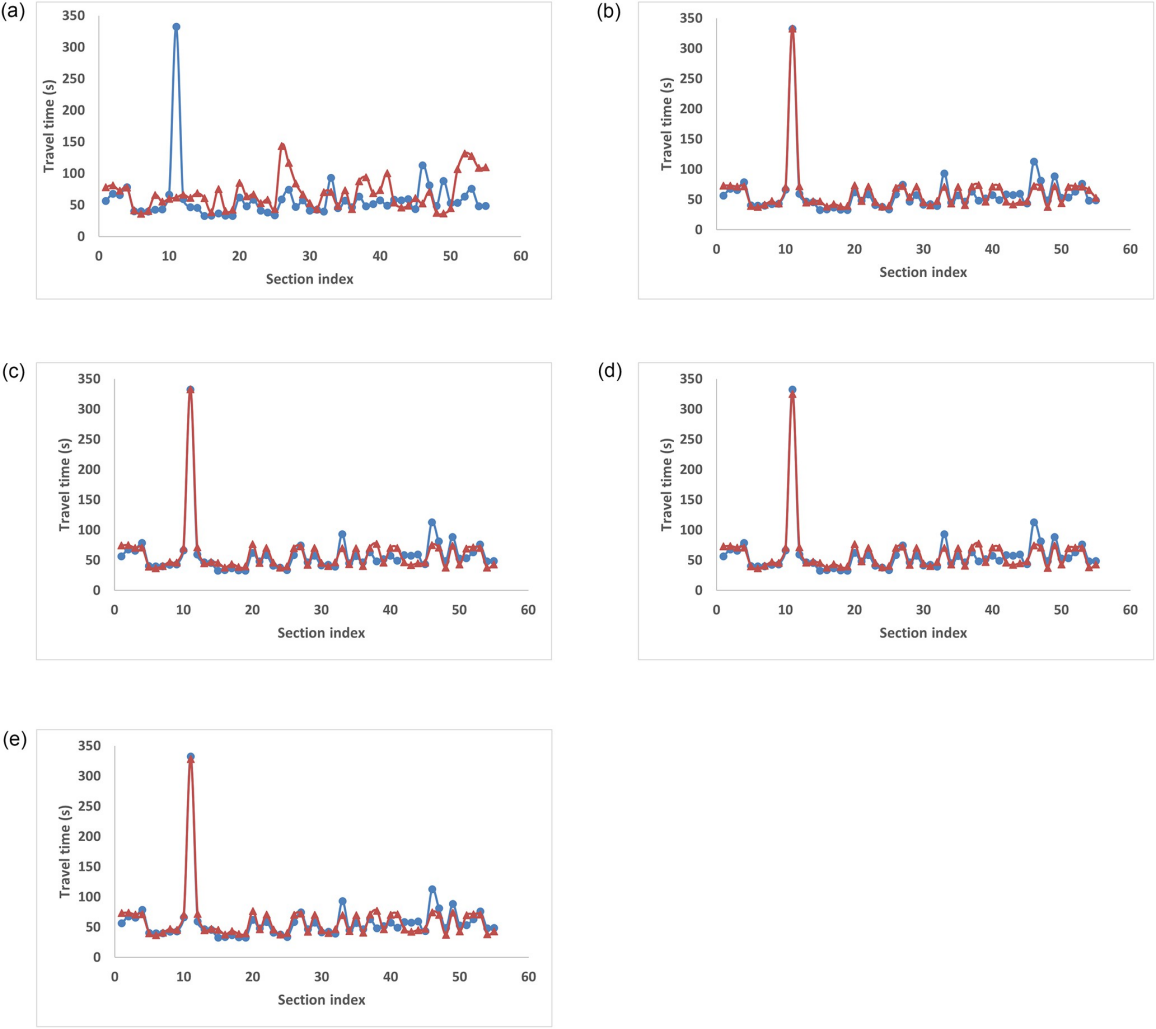
Measured and predicted travel times over iterations for the study stretch. (a) Iteration 0, (b) Iteration 1, (c) Iteration 2, (d) Iteration 3, (e) Iteration 4.

Fig 4 shows the variation of measured and predicted travel times for each section for a sample trip. Over iterations, the predictions better captured the variations in measured travel times, thereby reducing prediction errors. In iteration 0, where a single model was used on the entire training data set, the prediction errors were high, and the predictions failed to capture the patterns in the measured travel times. As iterations progressed, MAPE reduced, which shows that dividing the dataset into clusters and training multiple models on them is better than training a single model on the entire dataset. After *x* iterations, there was no significant change in the predictions, and hence the training was terminated. The *K* ANNs trained in this iteration were selected for further testing.

### Cluster membership for the *x*^th^ iteration

**Fig 5:**
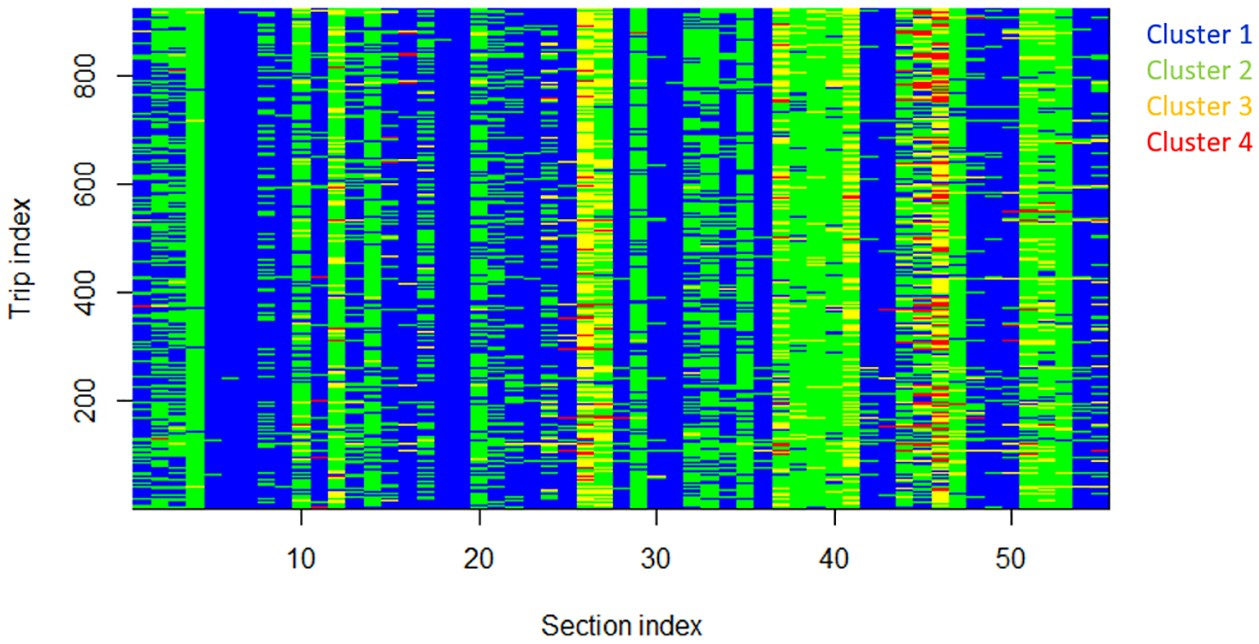
Cluster memberships for the *x*^th^ iteration.

Clustering is an unsupervised learning technique and has no associated labels. It is therefore challenging to evaluate the correctness of the partitions created. The clusters are commonly validated using visual inspection and prior knowledge about the system under consideration [37]. In this study, the clusters formed were analyzed manually to check if they corresponded to real-world traffic conditions. The clusters formed in the *x*^th^ iteration were analyzed to better understand the nature of clusters formed. Fig 5 shows the cluster memberships of travel times over time and space for the *x*^th^ iteration. The plot is color-coded to represent the cluster memberships. Data points that belong to the same cluster are shown with the same color. Using the Elbow method, the value of the optimum number of clusters *K* was found to be four. The four clusters are arranged in increasing order of average travel times as blue, green, yellow, and red, with blue indicating data points with least average travel times and red indicating data points with highest average travel times. Descriptive statistics of the four clusters are presented in Table 7.

**Table 7:** Descriptive statistics of clusters.

Cluster	Color	Number of members	Mean of travel times (s)	SD of travel times (s)	Range of travel times (s)
1	Blue	27643	42.54	8.96	[30, 75.38]
2	Green	18795	72.38	13.99	[40.90, 121.01]
3	Yellow	3614	146.94	34.39	[84.16, 244.45]
4	Red	713	339.91	98.12	[242.22, 1039.06]

The heat map shown in Fig 5 helps in identifying points that have similarities in travel time and predictability across time and space. From Table 7, cluster 1, which had the least average travel time across clusters, had more than 50% of the data points. Furthermore, most of the sections along the study stretch belonged to this cluster with the least average travel time for most times of the day. This indicates that these sections do not have much congestion for most times of the day. Cluster 4, on the other hand, had 1.41% of the data points and the highest average travel time. Cluster 4 usually occurred at bus stops and/or intersections along the study stretch. The presence of signals and bus stops increases delay and thereby increases travel time. For example, sections 45 and 46 belong to TIDEL park, a major intersection across the study stretch with a bus stop and two major intersections within 100 m distance. This section, as expected, belonged to clusters 3 and 4 with higher average travel times during most times of the day. Hence, it is concluded that meaningful clusters are formed on using both travel time and travel time prediction as feature vectors for clustering.

The clusters were further analyzed, both spatially and temporally. In the spatial analysis, the primary objective was to identify sections that exhibit similar behavior with respect to the magnitude of travel time and predictability. The sections across the study stretch were divided into two groups, namely low travel time and high travel time sections. A section was considered as belonging to a high travel time group if the majority of the trip travel times for that section were in the high travel time clusters (cluster 3 and cluster 4). A section was labeled low travel time section if the majority of the trip travel times belonged to the low travel time clusters (cluster 1 and cluster 2). It is seen that all the high travel time sections had either a bus stop or an intersection. These high travel time sections were similar with respect to the magnitude of travel times and ease of prediction. For example, section 26 (Sozhinganallur) was similar to section 47 (TIDEL park) because both belong to the high travel time group, compared to a section labeled as low travel time section.

**Fig 6:**
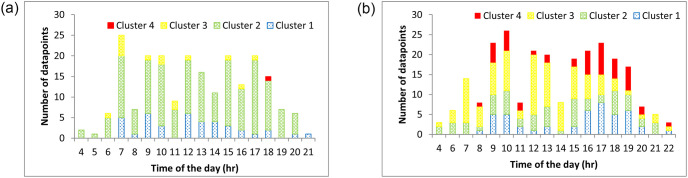
Variation of cluster memberships. (a) Low travel time section, (b) High travel time section.

To check for the temporal variations of the clusters formed, the cluster memberships were plotted for every hour of the day. Sample plots, one for a high travel time section and one for a low travel time section, are shown in Fig 6. Fig 6a shows the hourly variation of cluster membership for a low travel time section. In this case, most of the travel times belonged to the low travel time clusters. Fig 6b shows the hourly variation for a high travel time section. Here, the majority of the travel times belonged to the high travel time clusters (red and yellow bands). The number of points belonging to the red and yellow bands increased during the peak hours. As the evening peak set in, most trips were affected by the increasing congestion, resulting in higher travel times, leading to more membership in high travel time clusters.

**Fig 7:**
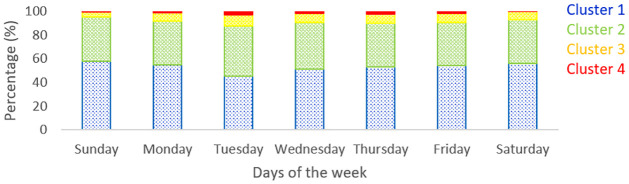
Proportion of memberships of clusters on each day of the week.

The next analysis concentrated on the proportion of clusters for each day of the week. For a sample week, the proportion of memberships of each cluster was calculated separately for each day of the week and plotted as shown in Fig 7. On all days, the maximum data points belonged to cluster 1, which is the lowest average travel time cluster. Furthermore, the number of data points belonging to the higher average travel time clusters 3 and 4 were higher during weekdays than weekends. This indicates that the travel times across the study stretch were higher on weekdays when the level of activity is high than on weekends when the level of activity on roads is low.

### Testing performance

**Fig 8:**
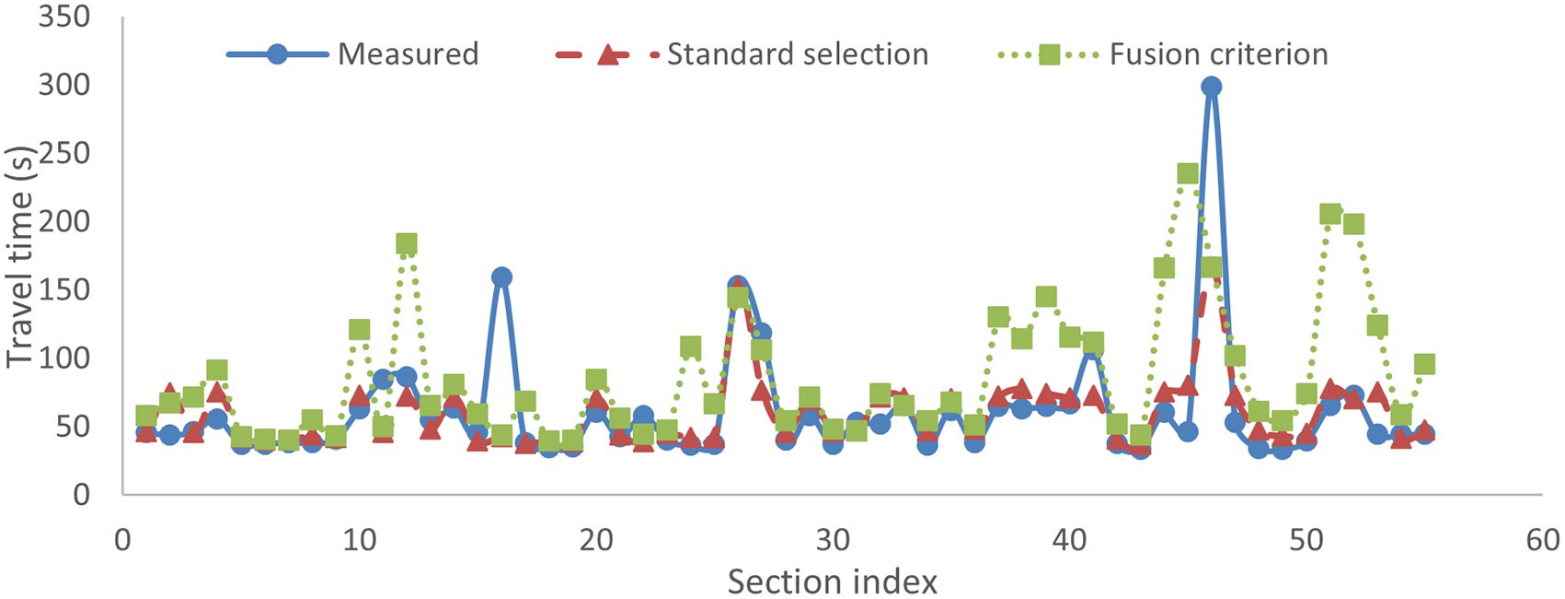
Measured and predicted travel times for a sample trip (standard selection vs. fusion criterion).

Once the training was over, the *K* ANNs were used for testing. Predictions were obtained using both selection and fusion criterion, as explained earlier. Fig 8 shows the measured and predicted travel time using both the standard selection criteria and fusion criteria. The prediction based on the standard selection criterion worked best in almost all cases, capturing the variations in measured travel time and yielding lower absolute error. The values of error metrics obtained for selection and fusion criterion respectively were: MAPE: 21.02%, 64.06%; MAE: 14.77s, 37.14s; and NRMSE: 0.1, 0.21. The selection-based prediction yielded better prediction accuracy, as seen from the values of all the three error indices. Hence, the standard selection-based criterion works better for predicting the travel times considering the spatiotemporal variations in the data compared to the fusion-based criterion.

**Fig 9:**
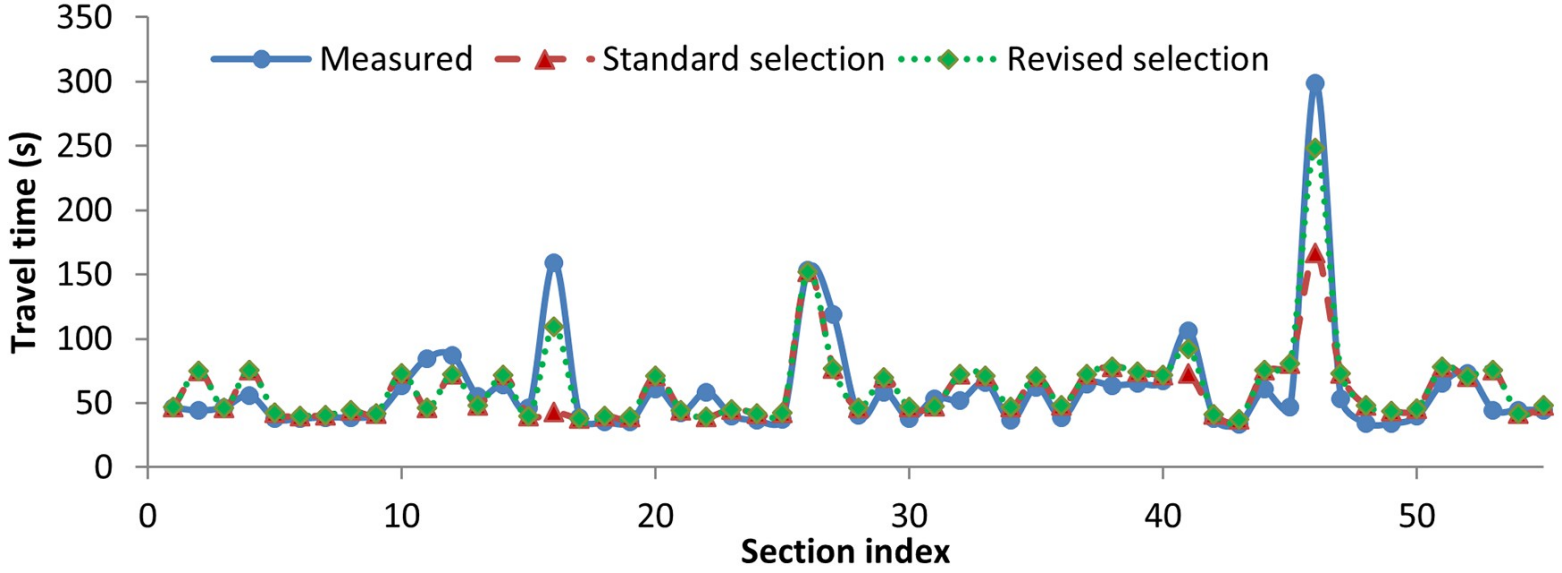
Measured and predicted travel times for a sample trip (standard selection vs. revised selection criterion).

The prediction errors were comparatively higher for sections belonging to the highest travel time cluster in most cases. To reduce the prediction errors for these sections, the standard selection-based criterion was revised using the revised selection-based criterion, as shown in Table 4. From Fig 9, it is seen that the revised selection could better capture the variations in the travel time data compared to the standard selection. The values of error metrics MAPE, MAE, and NRMSE obtained for the revised selection-based criterion were 17.02%, 12.34s, and 0.08, respectively. The revised selection-based criterion performed better than the standard selection-based criterion, as seen from the values of all three error indices. Hence, the revised selection-based prediction works best for predicting travel times considering the spatiotemporal variations in data. Further, error analysis was done using the results of prediction from the revised selection-based criterion.

**Fig 10:**
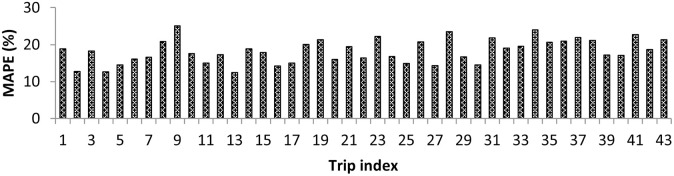
MAPE for all trips on a sample day.

**Fig 11:**
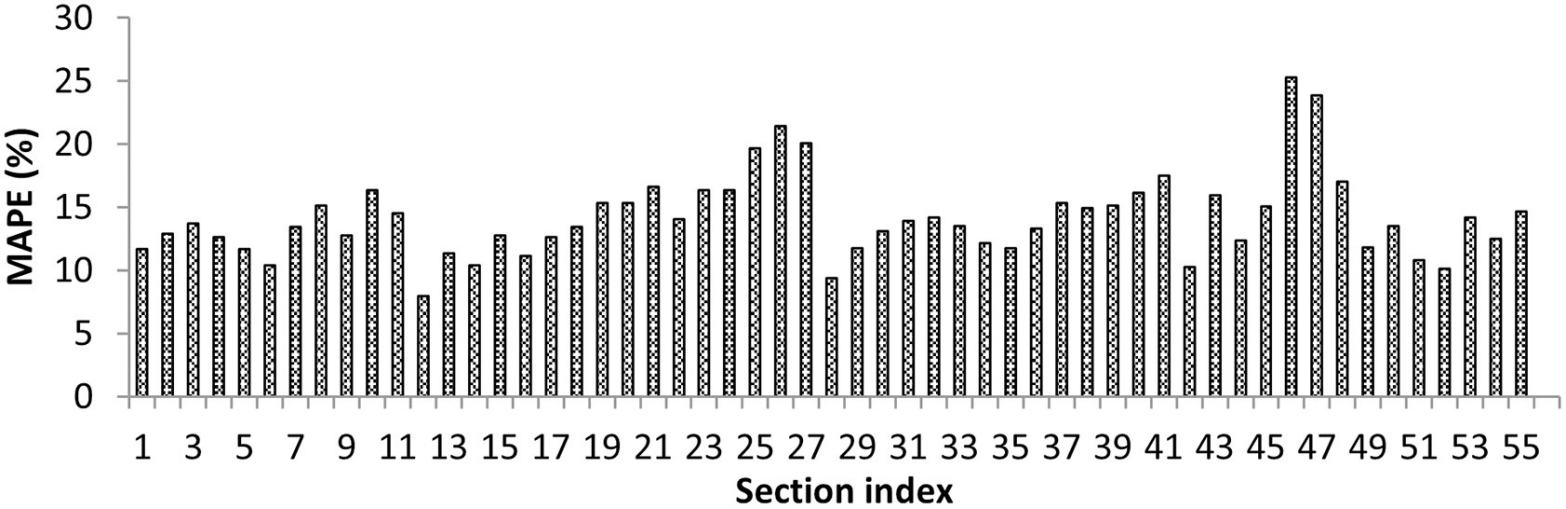
MAPE across sections on a sample day.

In the next step, trip level, section level, and day level errors were studied. Fig 10 shows the variation of MAPE using the revised selection-based criterion for all trips made on a sample day. The prediction errors were seen to vary from 12.4% to 25.1% for the proposed method. Next, MAPE values were plotted across sections for a sample day, as shown in Fig 11. The values ranged between 7.99% and 25.27% for the developed joint clustering and prediction framework. Next, day-level error values were studied. The daily MAPE values lay in the range of 17.3% to 20.7% for the study stretch, with Sunday having the lowest daily MAPE and Friday having the largest daily MAPE.

### Comparison of performance when only travel time is used as a feature vector for clustering

The effects of using both travel time and travel time prediction as feature vectors for clustering on predictions were examined by comparing the results obtained when only travel time is used for clustering. The same training procedure was repeated. Here, the optimum number of clusters was found to be five for *k*-means clustering, and the data set was divided into five clusters.

**Fig 12:**
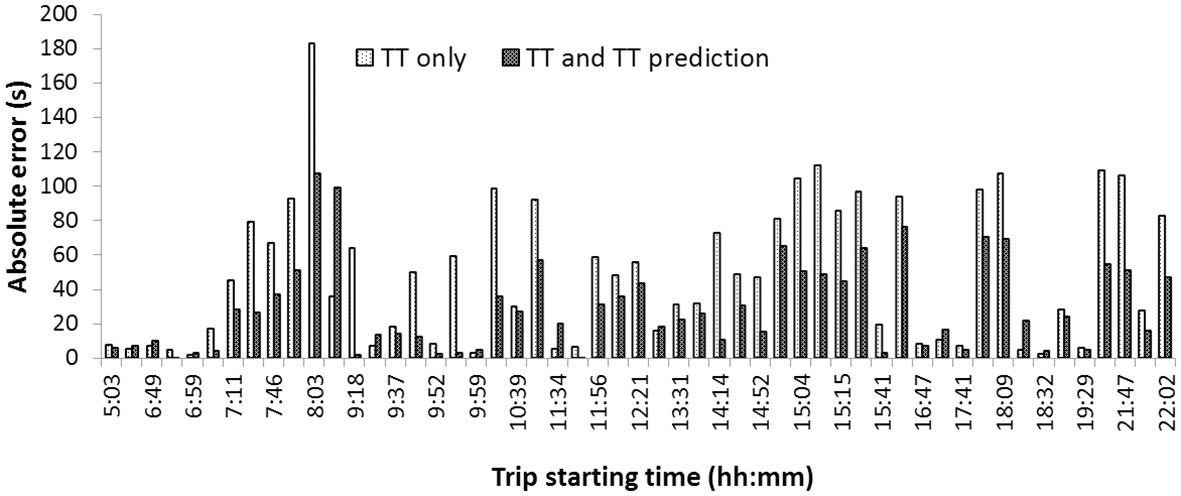
Effect of including prediction as a feature for clustering.

A comparison of predictions for a sample section with higher variance in travel times is shown in Fig 12. The predictions when both travel time and travel time predictions were used outperformed the case when only travel time was used as the feature vector. Hence, the choice of using both travel time and travel time predictions as feature vectors for clustering is justified as it significantly improves prediction accuracy.

### Performance comparison

**Fig 13:**
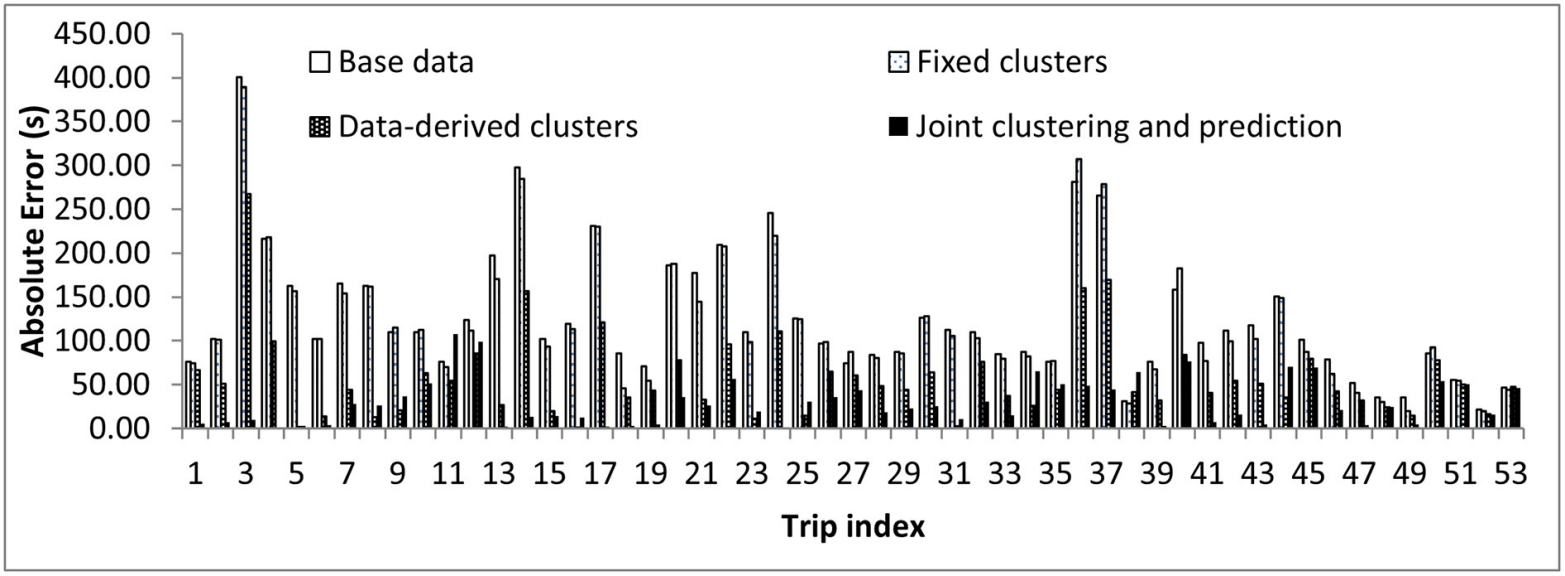
Absolute error for a sample section over time of the day.

The performance of the developed joint clustering and prediction approach was then compared with the three base methods. Fig 13 shows the results for a sample section over trips made on a weekday. With the use of clustering, prediction errors significantly reduced compared to the predictions on the base data and on the data with fixed clusters. The reduction in prediction errors was particularly evident for the peak hours of the day. Table 8 shows the MAPE values for the developed joint clustering and prediction approach, the three base methods discussed in the study, and a previous study reported on a similar data set by [1]. The predictions from the joint clustering and prediction approach outperformed all other prediction approaches. The predictions on the base data with no grouping had the highest error, indicating that patterns in the data play an important role in prediction techniques and must be taken into account. The errors for the case of the fixed clusters were also high because traffic patterns may not be static and may vary depending on the day, time, location, etc. For example, 09:00 am on Monday need not belong to a peak time with heavy traffic if a Monday is a holiday. The travel time patterns at 09:00 am on that day may be more like that of Sunday. Under such situations, considering fixed clusters of travel times into weekday peak, weekday off-peak, and weekend trips may result in the trips being wrongly grouped. Clustering algorithms can work efficiently under such situations and take into account the associated uncertainties. The superiority of the joint clustering and prediction framework over data-derived clusters confirms the earlier assumption that developing clusters based on the end-use (improving prediction accuracy in this study) results in better performance. The MAPE value reduced by 22.83% when the joint clustering and prediction framework was used for prediction compared to predictions using data-derived clusters, as seen from Table 8. The computation cost for different prediction approaches was also analyzed. It was seen that the time required to train the joint clustering and prediction framework was around five times when compared to the case of data-derived clusters. This is due to the many iterations required, as opposed to a single iteration in the case of predictions using data-derived clusters. However, this is justified as the developed framework brings in considerable improvement in prediction accuracy, and model retraining is required only when there is a significant change in traffic parameters. Hence, the developed joint clustering and prediction approach has a significant effect on improving the prediction accuracy by mining patterns from the data and using these patterns to make better predictions.

**Table 8:** MAPE values across different prediction approach.

Method	MAPE (%)
Base data	25.76
Fixed clusters	23.33
Data-driven clusters	18.4
Kumar et al., 2013 [1]	29.88
Joint clustering and prediction	14.2

Overall, the results showed the superiority of the proposed joint clustering and prediction framework. The MAPE decreased, and cluster memberships stabilized over iterations. Performance improvement compared to the base methodologies indicated that the proposed iterative clustering and prediction approach can considerably improve prediction accuracy.

## Summary and conclusions

In the present work, a joint clustering and prediction approach was developed for accurate prediction of bus travel times. It used an iterative process and considered the spatiotemporal patterns in traffic. The effects of integrating both clustering and prediction modules into an iterative joint clustering were analyzed. The impact of this integration on prediction accuracy was studied. The main findings and contributions of this study are summarized below.

An iterative joint clustering and prediction approach was developed to improve spatiotemporal pattern characterization and provide accurate bus travel time predictions. The sharp decrease in MAPE from iteration 0 to iteration 1 demonstrated the advantage of dividing the data set into clusters and building separate models on them, rather than building a single model on the entire dataset. It was observed that once the MAPE value stabilized, the cluster memberships also remained unchanged, indicating the formation of stable clusters. The clusters formed were analyzed to check if they constituted meaningful clusters. It was seen that sections with bus stops and/or intersections belonged to the higher average travel time clusters during most hours of the day.

A protocol to identify the correct cluster and prediction model for a test point was also developed. Two criteria—standard selection, and fusion-based criterion were incorporated. The predictions based on the selection strategy worked better than the latter. The predictions were further improved on using a revised selection-based criterion for high variance sections.

The idea of using both travel time and travel time predictions as feature vectors for clustering helped in grouping points that were similar in magnitude and predictability. The improvement in prediction accuracy justifies the selection of both feature vectors for clustering.

The developed joint clustering and prediction approach was compared with three base methods: a) on base data where no patterns or groups in the data are considered, b) data with fixed clusters based on chronological factors, and c) data-derived clusters. A significant decrease in prediction error was observed when the joint clustering and prediction approach was used, indicating its superiority over the other methods.

The present study proposed an umbrella framework that can be used for automated clustering and prediction of travel time data, which can also be used for prediction exercises regardless of the domain and the prediction model. The framework can be applied to predict variables that vary with both space and time. The joint prediction and clustering framework was able to provide accurate forecasts of bus travel times across the study stretch with highly varying travel times. Improvements in prediction accuracy can help provide accurate bus arrival information to passengers, thereby encouraging the use of public transportation among people.

The present study was based on GPS data from buses. Integrating the GPS data with other data sources such as weather data, area characteristics, the occurrence of accidents, social media alerts, etc., could improve prediction accuracy. In this study, equal-length sections were considered for analysis. However, most sections had similar travel times, as seen from the heat maps. Future studies could consider variable section lengths of uniform traffic characteristics and predict the travel times of similar sections with the same prediction model. The sections without bus stops and intersections would have similar ranges of travel times and can be considered a single section rather than multiple smaller sections. The differences or spikes may be evident only in the case of sections with bus stops and intersections, and such sections may have to be modeled separately. This may lead to more computationally efficient predictions and may be considered for future research.
